# Prevalence and Risk Factors of Postpartum Depression Among Women in Low-Income Developing Rural Areas: A Cross-Sectional Study in China

**DOI:** 10.1155/2024/8841423

**Published:** 2024-10-01

**Authors:** Mei Sun, Fanfan Cao, Jiayuan Peng, Jingfei Tang, Yuqing He, Yi Zeng, Xiangmin Tan, Qian Zhao

**Affiliations:** ^1^School of Nursing, Xinjiang Medical University, 567 Shangde North Road, Urumqi 830017, Xinjiang, China; ^2^Xiangya School of Nursing, Central South University, 172 Tongzipo Road Yuelu District, Changsha, Hunan 410013, China; ^3^School of Nursing, Changsha Medical University, 1501 Leifeng Avenue, Wangcheng District, Changsha 410219, Hunan, China; ^4^School of Rural Health, Monash University, 15 Sargeant Street, Warragul 3820, Victoria, Australia; ^5^School of Nursing, Anhui Medical University, 15 Feicui Road, Hefei 230601, Anhui, China

**Keywords:** intimate partner violence, postpartum depression, rural area

## Abstract

**Background:** Postpartum depression (PPD) significantly affects the welfare of mothers, infants, families, and communities. Mothers in rural areas often face low incomes, poor social security, low education levels, and inadequate medical services. These specific cultural, social, and economic aspects have led to a worsening of PPD in rural areas. However, the current situation of PPD among women in rural areas of China is still insufficiently explored.

**Aim:** This study aims to explore the prevalence and risk factors of PPD among women in low-income developing rural areas of China.

**Methods:** A cross-sectional design was used in this study. Edinburgh Postnatal Depression Scale (EPDS) was applied to evaluate PPD symptoms. General demographic questionnaire, obstetrics-/pediatrics-related questionnaire, and psychosocial-related questionnaire were adopted. Abuse Assessment Screen (AAS) was utilized to assess experienced intimate partner violence during pregnancy and postpartum. Social Support Rating Scale (SSRS) was utilized to measure their levels of social support.

**Results:** Of the 467 participants, the overall prevalence of PPD among women in rural areas of China was 16.5%, and the average EPDS score was 8.35 (SD = 4.50). PPD occurred most frequently at 7–9 months postpartum (33.8%). Six factors associated with PPD were whether the sex of the baby was in line with the family's expectations, monthly income of partners, social support, IPV during pregnancy and childbirth, and negative life events in the last 1 year, as well as physical and mental exhaustion from caring for a baby.

**Conclusions:** This study sheds light on the prevalence and various risk factors associated with PPD among women residing in low-income developing rural areas of China. The findings highlighted the need for targeted interventions and support systems designed to address the specific socioeconomic and cultural difficulties encountered by rural mothers.

## 1. Introduction

Postpartum depression (PPD) is a depressive state that usually occurs within 4 weeks to 1 year after delivery [1], which mainly shows persistent low or disturbed mood, depression, frustration, irritability, loss of appetite, sleep disorders, crying and even mental disillusionment, loss of confidence in life, and abandonment of the world, and the severe symptoms may include infanticide or suicide [1]. One of the highest risk periods for psychiatric disorders in women is after childbirth, and PPD is most common during this period [2]. A meta-analysis [3] showed that the global prevalence of PPD was found to be approximately 17.22%. And a report released by the Lancet Commission on 70 years of women's reproductive, maternal, newborn, child and adolescent health in China cites a systematic review that shows PPD affects at least one in 10 women in China, with prevalence rates ranging from 1.1% to 52.1% in different regions [4, 5].

Maternal living in rural areas is characterized by low incomes, poor social security, low education levels, inadequate medical services, and low levels of awareness of depression [6]. Moreover, rural areas in China are more susceptible to the traditional patriarchal/patriarchal ideology, in which, on the one hand, males are dominant in the family and society, and females have a relative lack of family and social support [7]; on the other hand, traditional preference for sons may put pressure on females to give birth [8]. Coupled with the fact that females will face major physical, psychological, and family structure changes after childbirth, all these factors may make mothers in rural areas more prone to PPD and may limit their help-seeking behaviors [7, 9] Thus, it is crucial to understand their PPD status and the factors influencing it at this particular and critical point in time.

The current situations of PPD among women in middle- to low-income country and rural areas are still insufficiently explored [3, 10]. The prevalence rate has been on the rise for many years, and the situation of PPD is even more serious in these regions due to increased levels of unemployment, poverty, internal conflict, and entrenched stereotypes [10–12]. An investigation conducted in the northern West Bank in Palestine revealed that the prevalence of PPD was 33.9%, and despite the impact of PPD, few studies have been conducted in Palestine [12]. A meta-analysis of 565 studies of PPD in 80 different countries or regions indicated that Southern Africa had the highest prevalence rate (39.96%) and there continues to be a significant lack of research from low- and middle-income or low-income developing countries [3]. The study highlights the need for further research in similar contexts across different global regions. More investigations in low-income settings can improve global understanding of PPD and inform targeted interventions to enhance the accessibility and affordability of mental health services, especially in underserved rural areas.

Most of the related studies in mainland China have been performed in cities and communities [13]. Despite the implementation of uniform mental health policies across urban and rural areas in China, there remains an uneven distribution of mental health resources [14]. Rural areas continue to face shortages of local mental health professionals, who are often burdened with heavy workloads and lack the time and resources to focus on this specific population [15]. The remoteness of the place of residence is also an important reason for the little attention they are paid [16, 17]. Currently, few studies have analyzed the factors influencing PPD among women in rural areas of China, and many factors have been reported to have an inconsistent conclusion on the effect of PPD [18, 19]. Studies on intimate partner violence (IPV) and PPD are still scarce in China, with more limited research on the impact of IPV throughout pregnancy and postpartum on PPD [20].

Thus, the aim and objectives of this study were to (i) investigate the prevalence of PPD on women in low-income developing rural areas of China and (ii) explore the influence of these significant demographic, obstetrics/pediatrics, and psychosocial factors (including IPV throughout pregnancy and postpartum) on PPD, providing a scientific empirical basis and reference direction for future PPD prevention, intervention, and protection policy development.

## 2. Methods

### 2.1. Study Design and Participants

During August 2020 to January 2021, a cross-sectional study was carried out in Jianghua Yao Autonomous County and Fenghuang County, Hunan Province, China. The inclusion criteria were (i) women of childbearing age (18–49 years) [21] and (ii) had given birth within the past 1 year. The exclusion criteria included (i) not permanent residents (living in these two counties for less than 6 months) and (ii) those who had difficulties reading and comprehending or had severe mental or psychological illnesses that would hinder their ability to respond to the questionnaire.

### 2.2. Instruments

General demographic questionnaire, obstetrics-/pediatrics-related questionnaire, and psychosocial-related questionnaire were developed by the research team based on a thorough review of the literature and consultation with experts. Then, participants were asked to complete the Edinburgh Postnatal Depression Scale (EPDS) [22], Abuse Assessment Screen (AAS) [23], and Social Support Rating Scale (SSRS) [24].

#### 2.2.1. General Demographic Questionnaire

The sociodemographic data include age, marital status, educational level, partner's educational level, being an only child, separated from partner during pregnancy and after childbirth, occupational status, partner's occupational status, monthly income, and partner's monthly income.

#### 2.2.2. Obstetrics/Pediatrics-Related Questionnaire

This section of the survey included number of pregnancies, number of children, whether the pregnancy was planned, weeks of gestation, type of delivery, whether regular obstetric examinations were conducted during pregnancy, whether an adverse pregnancy history was identified, whether complications or comorbidities during pregnancy were found, months postpartum, whether the baby had congenital disorders, whether the sex of the baby was in line with the family's expectations, and the type of feeding.

#### 2.2.3. Psychosocial-Related Questionnaire

The questionnaire included the couple's history of smoking and alcohol abuse, the couple's smoking and alcohol abuse during pregnancy, whether anything had happened during labor that worried the mother (e.g., prolonged labor, neonatal hypoxia, and fetal distress), whether caring for a baby was physically and emotionally exhausting, whether any negative life events had occurred in the last 1 year, attitudes toward domestic violence, whether witnessed domestic violence before 18 years of age, and whether experienced domestic violence before 18 years of age, and social support.

#### 2.2.4. EPDS

This scale was developed by Cox et al. [22]. It is a self-rating scale and has been translated and revised by Lee et al. [25] of the Chinese University of Hong Kong. It consists of 10 items rated 0–3 on a scale of 0–30, with higher scores indicating more severe PPD symptoms. Previous studies have shown that a cutoff value of 9/10 was better for sensitivity as a screen [26], but a cutoff value of 12/13 was better and more rigorous for assessing PPD [27], so a score of ≥13 was included in the PPD group in this study. The EPDS has a Cronbach's *α* coefficient of 0.837 and structural validity indicator KMO 0.881 of in the current study.

#### 2.2.5. AAS

The scale was developed by McFarlane et al. [23] to assess and screen for physical violence, psychological violence, obsessive–compulsive behavior, and fear of the perpetrator of violence in women during pregnancy or maternity. It was translated into Chinese by Leung et al. [28] at the University of Hong Kong in 1999, and Tiwari et al. [29] translated the scale into Chinese again in 2007 and tested the sensitivity and specificity of the scale. Comprising a total of nine items, the scale focuses on the exposure to physical or mental violence so far and the occurrence of physical, mental, or sexual violence within the past year and following pregnancy, as well as fear of the perpetrator. Subjects who answered “yes” to any of the items 3/4/5/6/7/8/9 were categorized as IPV-positive during pregnancy and postpartum. According to the findings of Moonesinghe, Rajapaksa, and Samarasinghe [30], this scale has good sensitivity (85.7%) and specificity (89.7%) for early screening and intervention. The AAS in this study has demonstrated a Cronbach's *α* coefficient of 0.770 and a KMO indicator of structural validity of 0.769.

#### 2.2.6. SSRS

The scale used in this study was developed by Xiao [24]. It was initially adapted from foreign scales and then modified based on the specific circumstances in China in 1986. The scale consists of 10 items, which are categorized into three dimensions: objective support (dimension 1, including items 2, 6, and 7), subjective support (dimension 2, including items 1, 3, 4, and 5), and social support utilization (dimension 3, including items 8, 9, and 10). This scale is used to assess the level of social support in the general population, with higher scores indicating higher levels of social support. In the present study, the Cronbach's *α* for internal consistency was 0.780, with a KMO measure of structural validity at 0.731. The scale also exhibited good test–retest reliability, with a coefficient of 0.92 [24].

### 2.3. Data Collection

Convenience sampling was used in this study. Since the target population of this study is mothers in rural areas, two national-level poor counties (Jianghua Yao Autonomous County and Fenghuang County in western Hunan Province), which are representative of Hunan Province, China, have a low level of economic development, and need to be economically alleviated, were selected for the survey.

The research team first contacted the leaders in charge of perinatal healthcare in each of the two counties. After fully communicating the purpose and content of the study and gaining understanding and support, the staff at perinatal healthcare assisted with the whole participant recruitment process. Recruitment efforts were undertaken through the display of recruitment posters, which detailed the study's purpose, content, risks, and benefits. After interested potential participants contacted the researchers, the details of this study were explained to them, and they were granted adequate time for contemplation regarding their involvement. Following the acquisition of participants' signed informed consent, questionnaires were dispensed. A uniform instruction term was used to guide the participants to fill in the questionnaire, which was collected on a one-to-one, face-to-face basis. To ensure the authenticity and validity of the data, the researchers retrieved the completed questionnaires from the participants on the spot and checked their completeness in a timely manner.

### 2.4. Ethical Approval and Considerations

This study was approved by the ethics committee of the University (Approval No. E202081). The study followed the informed consent guidelines, and the relevant investigations were conducted after obtaining the signed informed consent form from the participants. The researchers fully informed the participants about the purpose, procedure, and possible risks and benefits of this study. At the same time, participants were informed and understood that they held the right to terminate or withdraw from this study at any time. This study followed the principle of confidentiality, the participants filled out this questionnaire anonymously, and the data of this study were prohibited from appearing or divulging any personally identifiable information of the participants.

### 2.5. Data Analysis

The data analysis was performed using IBM SPSS statistics 20.0. Rates and component ratios were employed to describe count data. Means and standard deviations were used to describe continuous variables. In one-way analyses of variance, correlation analyses were conducted for continuous and hierarchical information, independent samples *t*-tests for dichotomous variables, and one-way ANOVA for unordered multicategorical variables. Variables with a significance level of *P*  < 0.05 were subsequently incorporated into a multiple linear regression model. All statistical analyses were performed with a significance level of *α* < 0.05 and two-sided probability (*P*-value).

## 3. Results

### 3.1. Participants' Sociodemographic Characteristics

In this study, a total of 475 questionnaires were sent out, and the total number of valid questionnaires was 467 with an effective response rate of 98.32%.  Table 1 shows the sociodemographic characteristics of the participants. The average age of the participants was 28.06 years (SD = 5.10). The majority of mothers were married (95.7%). 58.7% of mothers and 57.8% of their partners finished at least a high school education. Most of mothers were not the only child (416, 89.1%) and did not experience separation from their partners during pregnancy (335, 71.1%) and after childbirth (320, 68.5%). Regarding occupational status, 46.0% of mothers had occupations before pregnancy but not after delivery. 78.0% of mothers and 35.1% of their partners earn less than 3600 per month.

### 3.2. Prevalence of PPD

Of the 467 participants, the average EPDS score was 8.35 (SD = 4.50), and 77 (16.5%) had EPDS scores ≥13. The proportion of EPDS scores ≥13 in these four groups based on months postpartum were 23.4%, 19.5%, 33.8%, and 23.4% respectively (Table 2).

### 3.3. Factors Influencing PPD

#### 3.3.1. Sociodemographic Characteristics

A one-way test of sociodemographic characteristics showed that EPDS scores among women in rural areas were associated with age, marital status, separated from partner after childbirth, partner's occupational status, and partner's monthly income (*P* < 0.05) (Table 1).

#### 3.3.2. Obstetrics/Pediatrics Characteristics

Among obstetrics/pediatrics characteristics (Table 3), whether the pregnancy was planned, whether complications or comorbidities during pregnancy were found, whether the baby had congenital disorders, and whether the sex of the baby was in line with the family's expectations and type of feeding (*P* < 0.05) were found to be correlated with EPDS scores.

#### 3.3.3. Psychosocial Characteristics

EPDS scores differed significantly in mothers' history of smoking and alcohol abuse and smoking and alcohol abuse during pregnancy (Table 4). No significant associations were found between their partner's smoking history or alcohol abuse history and EPDS scores, but differences were observed between the partner's smoking during pregnancy (*t* = −3.201, *P*=0.001), their alcohol abuse during pregnancy (*t* = −2.836, *P*=0.005), and EPDS scores. The differences also exist among whether anything had happened during labor that worried the mother (e.g., prolonged labor, neonatal hypoxia, and fetal distress), whether caring for a baby was physically and emotionally exhausting, whether any negative life events had occurred in the last 1 year, mother's attitudes toward domestic violence, whether witnessed domestic violence before 18 years of age, whether experienced domestic violence before 18 years of age, and social support (*P* < 0.05).

#### 3.3.4. IPV

Among 467 mothers in rural areas, 82 of them (17.6%) reported IPV during pregnancy and postpartum, 74 (15.8%) reported psychosocial violence, 15 (3.2%) reported physical violence, and 20 (4.3%) reported sexual violence. The results of the chi-square test showed a significant difference (*P* < 0.001) between PPD and all types of IPV during pregnancy and postpartum (Table 5).

### 3.4. A Multiple Linear Regression Analysis of PPD

**Figure 1:**
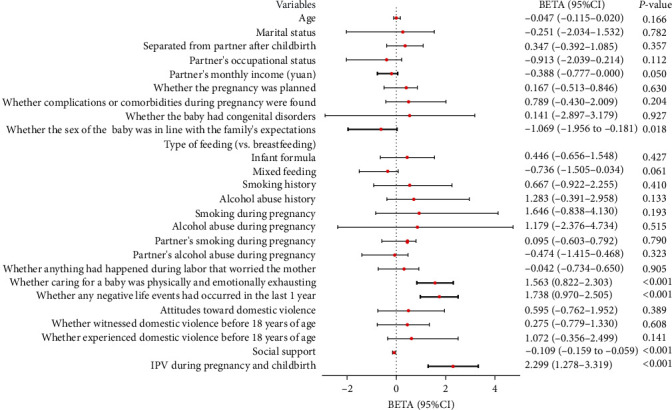
Factors influencing PPD: multiple linear regression. IPV: intimate partner violence.

The study utilized dummy variables to represent categorical variables and ranked data. Through multiple linear regression analysis, it was found that certain factors emerged as significant predictors of PPD among women in rural China (*F* = 11.818; *P* < 0.001; *R*^2^ = 0.401; adjusted *R*^2^ = 0.367), explaining 40.1% of the variance. The factors influencing PPD, in descending order of impact, were identified as follows: IPV during pregnancy and childbirth, whether any negative life events had occurred in the last 1 year, whether caring for a baby was physically and emotionally exhausting, whether the sex of the baby was in line with the family's expectations, partner's monthly income, and social support. The sex of the baby was in line with the family's expectations, monthly income of partners, and social support had positive effects on PPD, as illustrated in Figure 1. Conversely, IPV during pregnancy and childbirth, negative life events in the last 1 year, and physical and mental exhaustion from caring for a baby played negative roles.

## 4. Discussion

The results of this study showed that the average EPDS score was 8.35 (SD = 4.50), which is higher than the scores of mothers in cities but lower than those in other rural areas in China [10, 31]. To screen for the incidence of PPD, researchers have utilized a cutoff score of 13, and this threshold has been shown to be effective in detecting symptoms [27]. In this study, the proportion of PPD ≥ 13 among mothers in rural areas was 16.5%, which was higher compared to the prevalence among urban mothers (12%–15%) [32] and also higher than the average prevalence in mainland China (14.8%) [13]. It is generally in line with a previous study [8] and further confirms that mothers in rural areas suffer from a high prevalence of PPD. However, the prevalence in this study was lower than the reported rates of PPD in other rural areas (24.1%−25.34%) [8, 10]. One of the reasons is the different choice of cutoff values for screening PPD; some of the studies that used the EPDS to screen for PPD set the cutoff value at 9/10 points, and the incidence of PPD would have been higher. Secondly, the Chinese government's and hospitals' aggressive screening, prevention and control initiatives, and increasing emphasis on PPD in rural areas are also one of the major reasons [33].

After dividing participants equally into four groups based on month postpartum, we found that mothers 7–9 months postpartum in rural areas had the highest percentage of EPDS ≥ 13. This observation contrasts with the findings of O'Hara and McCabe [34], who reported that PPD occurred most frequently at 6 months postpartum. This discrepancy may be due to the heightened societal and familial expectations placed on mothers during the 7–9 months postpartum period in China. These expectations could lead to role conflicts and increased psychological burden, which might contribute to PPD. Moreover, traditional sense in China emphasizing the maternal role as the primary caregiver of the whole family could also exacerbate feelings of depression after delivery [35]. Furthermore, traditional cultural beliefs of “sitting the month” after childbirth in rural China, where mothers faced additional constraints like not to leave the house, not to bathe, and to minimize visits from relatives and friends, may exacerbate feelings of depression and unease, elevating the susceptibility to depression among mothers in rural areas 1 year postpartum [36]. Health workers can increase public awareness of mental health and PPD through media, newspapers, leaflets, and other channels. Emphasizing mental health education and promoting models of family and social support can further enhance the quality of healthcare accessible to rural mothers in China.

Among the sociodemographic factors, it is demonstrated that a higher partner's monthly income acts as a protective factor against PPD, aligning with previous studies [37]. In Chinese society, particularly within rural areas, traditional patriarchal values uphold the male as the familial leader, primarily responsible for financial obligations, while women are expected to support their husbands and raise their children. During the postpartum period, when women temporarily pause work and are emotionally vulnerable, the husband's income becomes crucial as the main source of family finances [37]. In this context, a low spousal income not only impacts daily household expenses but also contributes significantly to the financial responsibilities associated with newborn care, intensifying the adverse effects on maternal mental health. At present, China's maternity subsidies, medical expenses allowance, and childbirth insurance for rural women are gradually being refined, and attention still needs to be paid in the future to the rights and guarantees of this population [38]. Policymakers should improve social assistance by enhancing the social security system, increasing regular assessments for maternal depression and related mental health issues, and including the costs of diagnosing and managing maternal depression in maternity insurance coverage. Additionally, the state should boost ideological and political education in remote rural areas, eliminate gender discrimination in employment, and promote equal job opportunities. Relevant departments should provide job placements and vocational training for rural women, as well as maternity leave for those with reproductive needs, to support women's full potential.

The present study revealed that whether the sex of the baby was in line with the family's expectations was significantly associated with PPD in rural areas in China, which is consistent with the findings of several previous studies [8, 39]. The disparity between the gender of the newborn and the anticipated gender plays a significant role in the onset of PPD, potentially influenced by Chinese traditional ideologies of favoring male offspring, particularly prevalent in rural areas where a strong patriarchal belief system exists. The discrepancy between the expected birth of a son and the actual delivery of a daughter can lead to diminished self-worth and low self-esteem among mothers, ultimately contributing to PPD [8]. However, the governmental stance on gender equality and the protection of women's and children's rights highlights the need to bridge the gaps in development experienced by rural women facing PPD due to gender-related expectations [40]. The Chinese Women's Development Program (2021–2030) acknowledges discrimination against women, emphasizes the need for gender equality promotion, and advocates for a supportive social environment to advance women's rights [40]. The implementation of gender equality measures is critical to enhancing maternal and child health and mental health in rural areas of China and developing countries with similar cultural backgrounds.

Other obstetric and pediatric characteristics did not show significance in the multiple linear regression analysis, which contrasts with findings from other studies [39, 41]. For instance, one study found higher prevalence of PPD among multiparous women compared to primiparous women, while another study reported the opposite result [42, 43]. Further research is needed to empirically support the relationship between number of pregnancies and PPD among rural Chinese mothers. Delivery type has been identified as a factor related to PPD, with prolonged labor, cesarean section, and preterm delivery increasing the risk in China [44]. Cheng [45] also identified irregular obstetric care during pregnancy as a risk factor for PPD among mothers in impoverished areas. The lack of significance of these factors in our study may be attributed to the positive impact of China's healthcare policies, including guidelines updated in 2011 and 2018, which have improved prenatal care access and quality [46]. With the continuous promotion of healthcare services for the entire childbirth process, pregnant women can have five free prenatal checkups, and their rate of prenatal checkups has risen. The 2019 China Maternal and Child Health Development Report noted that the national prenatal checkup rate has steadily increased, from 83.7% in 1996 to 96.6% in 2018 and from 80.6% to 95.8% in rural areas [33]. And 95.5% of the participants in this study had a regular prenatal checkup. At the same time, the incidence of comorbidities and complications during pregnancy was smaller and detected in a timely manner; the health of fetuses and newborns was safeguarded [33]. To further enhance maternal mental health, policymakers could consider implementing a comprehensive maternity screening and intervention model. This model would include depression screening during obstetric care, interventions by maternal and child healthcare institutions, and consultations or treatments provided by mental health specialists or institutions.

It has been observed that physical and mental exhaustion from caring for a baby constitutes a significant risk factor for PPD among rural mothers. This finding contrasts with Zhang [47]'s study but aligns with the majority of other research [48, 49]. Evidence indicates that women experiencing physical and mental exhaustion from baby care are 8.015 times more likely to develop PPD compared to those who do not [49]. Postbirth, mothers often face challenges such as frequent infant crying, sleep deprivation, and feeding difficulties, contributing to their exhaustion [48]. In rural China, partners typically need to work immediately after childbirth to support the family financially, leaving little time for providing real-time support to mothers. Traditional gender norms may also constrain the participation of men in families, particularly in caring for infants and mothers, further isolating mothers and exacerbating their fatigue, anxiety, and sense of helplessness, thereby increasing PPD risk [20]. To address these challenges, it is crucial for partners and family members to change misconceptions about sharing responsibility for infant care and to offer mothers adequate support and companionship postchildbirth. Healthcare professionals can play a vital role by assessing mothers' physical and mental health during postnatal checkups, providing health education, enhancing postpartum care visits, and educating partners about the specific difficulties faced by rural mothers.

Consistent with previous literature, the results showed that negative life events in the last 1 year were a relatively important predictor of PPD among mothers in rural areas [50]. In this study, negative life events in the last 1 year were defined as life events that caused stress, distress, or strain on the mother's mental well-being in the last 1 year. Pregnancy and childbirth are inherently stressful events for the mothers, and the occurrence of negative life events increases feelings of stress, tension, and threat, and the accumulation of these negative emotions can induce and promote PPD [20]. Wen [51] indicated that negative life events as stressors have a significant impact on the development of PPD, but high levels of social support and effective coping and management can prevent or stop the occurrence of PPD. Thus, healthcare providers should help mothers to face negative life events and the stress and difficulties of childbirth and infant rearing positively and teach them techniques to relieve stress and cope with problems; at the same time, they should provide adequate health education, informing them of the adverse effects of negative responses on PPD and reminding their family members of the importance of fully supports.

High level of social support is a protective factor for PPD, and this result is in line with the findings of Yang, Zhang, and Yu [9]. Faisal-Cury et al. [52] demonstrated that a high level of social support is not only a protective factor for PPD, but also has a relatively independent effect on it. From the beginning of pregnancy to 1 year after delivery in China, during which mothers devote most of their time and energy to waiting for and caring for their babies, they are forced to detach themselves from some of their social activities and social relationships, and this change in their normal social interactions may make them feel exhausted and anxious [35]. When negative events that threaten human health are taken as stressors, social support can reduce the adverse physiological and psychological effects by improving people's appraisal of the events and enhancing their feelings of support and belonging [39]. Therefore, policymakers should integrate PPD prevention and treatment into essential public services, offering free preventive support to enhance social support for rural mothers. Healthcare providers can monitor mothers' mental health through community hospitals and phone check-ins. Additionally, strengthening psychological helplines is crucial to provide counseling, guidance, and appropriate referrals for mothers showing signs of depression.

In this study, IPV during pregnancy and childbirth was identified as the most important risk factor of PPD among mothers in rural areas of China. Multiple studies have also found a significant positive association between IPV and PPD [53]. Mothers who experience IPV have a two- to threefold higher risk of developing PPD than those who do not [54]. As the frequency and type of IPV increases, the incidence and severity of PPD increase [55, 56]. Research in China on the occurrence of IPV throughout the entire course of pregnancy and labor in relation to PPD is very limited. The close relationship between PPD and IPV still needs to be explored in depth in the future. And it has been stated that psychological violence is not only a strong risk factor for PPD, but also an independent risk factor [57, 58]. Partner sexual violence during pregnancy and other forms of IPV have also been identified as contributing factors to the development of PPD [56]. A history of past sexual abuse is also a predisposing factor for PPD [55]. In China, public awareness of the prevention of and opposition to domestic violence has increased with the enactment of the Law against Domestic Violence, and these changes may have diminished the propensity of men to commit physical and sexual violence [59]. However, while the status of women has improved, conflicts over the social resources of men and women, especially in terms of socioeconomic status, and conflicts with traditional patriarchal/husbandly power concepts still give rise to violence between intimate partners [56]. Therefore, it is recommended that healthcare professionals pay attention to assessing whether mothers are suffering from IPV, especially psychological violence, during routine pregnancy and delivery checkups, and provide timely psychological support and, if necessary, report it to the public security system or make a referral.

## 5. Limitations

In this study, firstly, the participants were limited to women within 1 year postpartum in two poor counties (Jianghua and Fenghuang counties) in Hunan Province. However, these two districts are relatively representative in terms of economic level. Secondly, the study may exist with recall bias and reporting bias. Particularly among women in rural China, reluctance to disclose experiences of PPD and IPV from spouses or family members may stem from traditional cultural expectations of “keeping the shame out of the family” [60]. Thirdly, these conditions would lead to an underestimation of the prevalence of PPD in this population. Finally, other unexplored factors should be investigated through a national multicenter, large-sample cross-sectional survey and in-depth qualitative interviews. For example, physical activity during pregnancy has been shown to have a significant negative correlation with PPD in previous studies [61, 62]. Long-term impacts of relevant factors should be explored through longitudinal studies. Additionally, targeted intervention programs, including implementation research, should be developed to mitigate the effects of PPD and enhance the welfare of mothers, infants, and families in these specific communities.

## 6. Conclusion

In this study, we explored PPD and its influencing factors among mothers in rural China, which helps to promote the attention of academics, clinicians, and policy makers to the PPD status of the population in rural regions and other developing countries. The prevalence of PPD among this population is high, and the findings underscore the significant impact of socioeconomic and cultural factors such as low income, gender inequality perceptions, and limited social support on the occurrence of PPD in these communities. Moreover, IPV during pregnancy and postpartum, negative life events, and the physical and mental strain of caring for an infant emerged as substantial contributors to PPD among rural women. Future prevention and interventions need to be culturally sensitive and adapted to local contexts to ensure acceptance and effectiveness. And longitudinal studies could be further used to explore changes in PPD, IPV, and psychosocial factors over time in rural areas.

## Figures and Tables

**Table 1:** Sociodemographic characteristics of participants and their effect on PPD (*N* = 467).

Sociodemographic factors	$\overset{―}{x}$ ± *s/n* (%)	EPDS score ($\overset{―}{x}$ ± *s*)	*t/F/r*	*P*
Age	28.06 ± 5.10	8.35 ± 4.50	−0.012	0.012*⁣*^*∗*^
Marital status			2.345	0.019*⁣*^*∗*^
Single	20 (4.3)	10.65 ± 4.67	—	—
Married	447 (95.7)	8.25 ± 4.47	—	—
Education level			1.771	0.152
Junior high and below	193 (41.3)	8.70 ± 4.54	—	—
High school/technical school	124 (26.6)	8.20 ± 4.25	—	—
Professional training college	72 (15.4)	8.71 ± 4.66	—	—
Undergraduate and above	78 (16.7)	7.40 ± 4.56	—	—
Partner's educational level			1.661	0.175
Junior high and below	197 (42.2)	8.76 ± 4.55	—	—
High school/technical school	141 (30.2)	8.42 ± 4.47	—	—
Professional training college	69 (14.8)	7.45 ± 4.33	—	—
Undergraduate and above	60 (12.8)	7.92 ± 4.52	—	—
Being an only child			−1.194	0.237
No	416 (89.1)	8.25 ± 4.37	—	—
Yes	51 (10.9)	9.20 ± 5.45	—	—
Separated from partner during pregnancy			0.220	0.826
No	335 (71.7)	8.38 ± 4.36	—	—
Yes	132 (28.3)	8.28 ± 4.85	—	—
Separated from partner after childbirth			−2.494	0.013*⁣*^*∗*^
No	320 (68.5)	7.98 ± 4.13	—	—
Yes	147 (31.5)	9.18 ± 5.13	—	—
Occupational status			1.583	0.193
No occupation before pregnancy and after birth	97 (20.8)	8.06 ± 4.23	—	—
Prepregnancy nonoccupational and postpregnancy occupational	8 (1.7)	8.00 ± 7.95	—	—
Occupation before pregnancy and no occupation after birth	215 (46.0)	8.84 ± 4.35	—	—
Occupation before pregnancy and after birth	147 (31.5)	7.86 ± 4.63	—	—
Partner's occupational status			2.899	0.004*⁣*^*∗*^
No	49 (10.5)	10.10 ± 5.14	—	—
Yes	418 (89.5)	8.15 ± 4.38	—	—
Monthly income (yuan)			−0.034	0.458
Less than 1800	274 (58.7)	8.39 ± 4.34	—	—
1801–3600	90 (19.3)	8.87 ± 5.12	—	—
3601–6000	79 (16.9)	7.65 ± 4.40	—	—
6001 or more	24 (5.1)	8.33 ± 4.17	—	—
Partner's monthly income (yuan)			−0.151	0.001*⁣*^*∗*^
Less than 1800	37 (7.9)	10.00 ± 5.01	—	—
1801–3600	127 (27.2)	8.95 ± 4.68	—	—
3601–6000	174 (37.3)	8.10 ± 4.22	—	—
6001 or more	129 (27.6)	4.40 ± 0.39	—	—

*⁣* ^*∗*^Statistically significant.

**Table 2:** EPDS scores in four groups (*N* = 467).

Groups	EPDS score ($\overset{―}{x}$ ± *s*)	EPDS scores ≥ 13, *n* (%)	EPDS scores < 13, *n* (%)	Total *n* (%)
G1 (1–3 months after delivery)	7.94 ± 4.00	18 (23.4)	143 (36.7)	161 (34.5)
G2 (4–6 months after delivery)	8.01 ± 3.85	15 (19.5)	92 (23.6)	107 (22.9)
G3 (7–9 months after delivery)	9.01 ± 4.96	26 (33.8)	85 (21.8)	111 (23.8)
G4 (10–12 months after delivery)	8.70 ± 5.36	18 (23.4)	70 (17.9)	88 (18.8)

**Table 3:** Obstetrics/pediatrics characteristics of participants and their effect on PPD (*N* = 467).

Obstetrics/pediatrics characteristics	*n* (%)/$\overset{―}{x}$ ± *s*	EPDS score ($\overset{―}{x}$ ± *s*)	*t/F/r*	*P*
Number of pregnancies			0.035	0.452
1	138 (29.6)	8.54 ± 4.95	—	—
2	167 (35.7)	7.69 ± 4.06	—	—
3 or more	162 (34.7)	8.88 ± 4.47	—	—
Number of children			0.016	0.730
1	205 (43.9)	8.45 ± 4.97	—	—
2	204 (43.7)	8.04 ± 3.89	—	—
3 or more	58 (12.4)	9.10 ± 4.72	—	—
Whether the pregnancy was planned			2.076	0.038*⁣*^*∗*^
No	242 (51.8)	8.77 ± 4.80	—	—
Yes	225 (48.2)	7.91 ± 4.12	—	—
Weeks of gestation	38.75 ± 2.16	8.35 ± 4.50	0.022	0.630
Type of delivery			0.978	0.329
Natural births	272 (58.2)	8.53 ± 4.47	—	—
Cesarean section	195 (41.8)	8.11 ± 4.54	—	—
Whether regular obstetric examinations were conducted during pregnancy			2.021	0.056
No	21 (4.5)	10.67 ± 5.41	—	—
Yes	446 (95.5)	8.24 ± 4.43	—	—
Whether an adverse pregnancy history was identified			−1.167	0.244
No	353 (75.6)	8.22 ± 4.56	—	—
Yes	114 (24.4)	8.78 ± 4.31	—	—
Whether complications or comorbidities during pregnancy were found			−2.652	0.008*⁣*^*∗*^
No	426 (91.2)	8.18 ± 4.41	—	—
Yes	41 (8.8)	10.12 ± 5.06	—	—
Months postpartum	6 (3, 8)	8.35 ± 4.50	0.048	0.304
Whether the baby had congenital disorders			−2.427	0.016*⁣*^*∗*^
No	460 (98.5)	8.29 ± 4.48	—	—
Yes	7 (1.5)	12.43 ± 4.50	—	—
Whether the sex of the baby was in line with the family's expectations			4.026	＜0.001*⁣*^*∗*^
No	85 (47.5)	10.28 ± 5.01	—	—
Yes	382 (50.1)	7.92 ± 4.27	—	—
Type of feeding			3.945	0.020*⁣*^*∗*^
Breastfeeding	289 (61.9)	8.41 ± 0.27	—	—
Infant formula	52 (11.1)	9.71 ± 0.72	—	—
Mixed feeding	126 (27.0)	7.66 ± 0.36	—	—

*⁣* ^*∗*^Statistically significant.

**Table 4:** Psychosocial characteristics of participants and their effect on PPD (*N* = 467).

Psychosocial characteristics	$\overset{―}{x}$ ± *s/n* (%)	EPDS score ($\overset{―}{x}$ ± *s*)	*t*/*r*	*P*
Smoking history			−3.701	0.001*⁣*^*∗*^
No	423 (90.6)	8.07 ± 4.34	—	—
Yes	44 (9.4)	11.05 ± 5.14	—	—
Alcohol abuse history			−4.357	＜0.001*⁣*^*∗*^
No	433 (92.7)	8.10 ± 4.37	—	—
Yes	34 (7.3)	11.53 ± 4.98	—	—
Smoking during pregnancy			−3.201	0.001*⁣*^*∗*^
No	455 (97.4)	8.25 ± 4.44	—	—
Yes	12 (2.6)	12.42 ± 5.11	—	—
Alcohol abuse during pregnancy			−2.638	0.009*⁣*^*∗*^
No	462 (98.9)	8.30 ± 4.48	—	—
Yes	5 (1.1)	13.6 ± 3.85	—	—
Partner's smoking history			-−0.400	0.689
No	166 (35.5)	8.24 ± 4.28	—	—
Yes	301 (64.5)	8.42 ± 4.62	—	—
Partner's alcohol abuse history			−1.081	0.281
No	353 (75.6)	8.22 ± 4.42	—	—
Yes	114 (24.4)	8.76 ± 4.73	—	—
Partner's smoking during pregnancy			−3.201	0.001*⁣*^*∗*^
No	223 (47.8)	8.25 ± 4.44	—	—
Yes	244 (52.2)	12.42 ± 5.11	—	—
Partner's alcohol abuse during pregnancy			−2.836	0.005*⁣*^*∗*^
No	377 (80.7)	8.05 ± 4.38	—	—
Yes	90 (19.3)	9.62 ± 4.80	—	—
Whether anything had happened during labor that worried the mother (e.g., prolonged labor, neonatal hypoxia, and fetal distress)			−3.651	＜0.001*⁣*^*∗*^
No	251 (53.7)	7.66 ± 4.33	—	—
Yes	216 (46.3)	9.16 ± 4.57	—	—
Whether caring for a baby was physically and emotionally exhausting			−7.814	＜0.001*⁣*^*∗*^
No	204 (43.7)	6.65 ± 3.83	—	—
Yes	263 (56.3)	9.68 ± 4.54	—	—
Whether any negative life events had occurred in the last 1 year			−9.038	＜0.001*⁣*^*∗*^
No	273 (58.5)	6.85 ± 3.76	—	—
Yes	194 (41.5)	10.47 ± 4.61	—	—
Attitudes toward domestic violence			−4.505	＜0.001*⁣*^*∗*^
Disagree	430 (92.1)	8.08 ± 4.35	—	—
Agree	37 (7.9)	11.49 ± 5.10	—	—
Whether witnessed domestic violence before 18 years of age			−2.819	0.006*⁣*^*∗*^
No	395 (84.6)	8.07 ± 4.31	—	—
Yes	72 (15.4)	9.90 ± 5.20	—	—
Whether experienced domestic violence before 18 years of age			−3.922	＜0.001*⁣*^*∗*^
No	427 (91.4)	8.07 ± 4.33	—	—
Yes	40 (8.6)	11.40 ± 5.21	—	—
Social support	40.04 ± 7.41	8.35 ± 4.50	−0.385	＜0.001*⁣*^*∗*^
Objective support	8.89 ± 2.84	8.35 ± 4.50	−0.189	＜0.001*⁣*^*∗*^
Subjective support	23.71 ± 4.71	8.35 ± 4.50	−0.363	＜0.001*⁣*^*∗*^
Social support utilization	7.44 ± 1.92	8.35 ± 4.50	−0.315	＜0.001*⁣*^*∗*^

*⁣* ^*∗*^Statistically significant.

**Table 5:** IPV during pregnancy and childbirth of participants and their effect on PPD (*N* = 467).

IPV during pregnancy and childbirth	*n* (%)	EPDS score ($\overset{―}{x}$ ± *s*)	*t*	*P*
IPV during pregnancy and childbirth			−8.331	＜0.001*⁣*^*∗*^
No	385 (82.4)	7.51 ± 3.94	—	—
Yes	82 (17.6)	12.3 ± 4.88	—	—
Intimate partner psychological violence			−8.163	＜0.001*⁣*^*∗*^
No	393 (81.2)	7.58 ± 3.97	—	—
Yes	74 (15.8)	12.49 ± 4.88	—	—
Intimate partner physical violence			−4.693	＜0.001*⁣*^*∗*^
No	452 (96.8)	8.18 ± 4.43	—	—
Yes	15 (3.2)	13.60 ± 3.46	—	—
Intimate partner sexual violence			−5.719	＜0.001*⁣*^*∗*^
No	447 (95.7)	8.11 ± 4.36	—	—
Yes	20 (4.3)	13.80 ± 4.14	—	—

Abbreviation: IPV, intimate partner violence.

*⁣* ^*∗*^Statistically significant.

## Data Availability

Data are available on request from the authors.
